# Impact of COVID-19 response on global surgical volumes: an ongoing observational study

**DOI:** 10.2471/BLT.20.264044

**Published:** 2020-09-03

**Authors:** Vikas N O’Reilly-Shah, Wil Van Cleve, Dustin R Long, Vanessa Moll, Faye M Evans, Jacob E Sunshine, Nicholas J Kassebaum, Ewen M Harrison, Craig S Jabaley

**Affiliations:** a Department of Anesthesiology & Pain Medicine, University of Washington School of Medicine, RR450, 1959 NE Pacific St, Seattle, Washington 98195, United States of America (USA).; b Institute for Health Metrics and Evaluation, University of Washington, Seattle, USA.; c Institute of Anesthesiology, University Hospital Zurich, Zurich, Switzerland.; d Department of Anesthesiology, Critical Care and Pain Medicine, Harvard Medical School, Boston, USA.; e Usher Institute, University of Edinburgh, Edinburgh, Scotland, United Kingdom.; f Department of Anesthesiology, Emory University School of Medicine, Atlanta, USA.

## Abstract

**Objective:**

To determine whether location-linked anaesthesiology calculator mobile application (app) data can serve as a qualitative proxy for global surgical case volumes and therefore monitor the impact of the coronavirus disease 2019 (COVID-19) pandemic.

**Methods:**

We collected data provided by users of the mobile app “Anesthesiologist” during 1 October 2018–30 June 2020. We analysed these using RStudio and generated 7-day moving-average app use plots. We calculated country-level reductions in app use as a percentage of baseline. We obtained data on COVID-19 case counts from the European Centre for Disease Prevention and Control. We plotted changing app use and COVID-19 case counts for several countries and regions.

**Findings:**

A total of 100 099 app users within 214 countries and territories provided data. We observed that app use was reduced during holidays, weekends and at night, correlating with expected fluctuations in surgical volume. We observed that the onset of the pandemic prompted substantial reductions in app use. We noted strong cross-correlation between COVID-19 case count and reductions in app use in low- and middle-income countries, but not in high-income countries. Of the 112 countries and territories with non-zero app use during baseline and during the pandemic, we calculated a median reduction in app use to 73.6% of baseline.

**Conclusion:**

App data provide a proxy for surgical case volumes, and can therefore be used as a real-time monitor of the impact of COVID-19 on surgical capacity. We have created a dashboard for ongoing visualization of these data, allowing policy-makers to direct resources to areas of greatest need.

## Résumé

**Objectif:**

Déterminer si les données provenant d'applications mobiles de calcul dédiées à l'anesthésie et munies d'une fonction de géolocalisation peuvent servir de substitut en vue d'évaluer le nombre d'interventions chirurgicales dans le monde et, par conséquent, de mesurer l'impact de la pandémie de maladie à coronavirus 2019 (COVID-19) sur ces interventions.

**Méthodes:**

Nous avons récolté les données fournies par les utilisateurs de l'application mobile «Anesthesiologist» entre le 1^er^ octobre 2018 et le 30 juin 2020. Nous les avons analysées à l'aide du logiciel RStudio et avons généré des graphiques représentant la moyenne mobile d'utilisation de l'application sur une période de 7 jours. Nous avons calculé les baisses d'utilisation de l'application au niveau national en guise de pourcentage de référence. Nous nous sommes procuré les informations concernant le nombre de cas de COVID-19 auprès du Centre européen de prévention et de contrôle des maladies. Enfin, nous avons représenté sous forme de graphique les variations d'utilisation de l'application ainsi que l'évolution du nombre de cas de COVID-19 dans une série de pays et régions.

**Résultats:**

Au total, 100 099 utilisateurs originaires de 214 pays et régions nous ont communiqué leurs données. Nous avons observé une diminution dans l'utilisation de l'application durant les vacances, les week-ends et la nuit, ce qui correspond aux fluctuations prévues en matière de volume d'interventions. Nous avons également constaté que l'apparition de la pandémie avait entraîné une baisse considérable de l'utilisation de l'application. Nous avons noté une importante corrélation croisée entre le nombre de cas de COVID-19 et cette baisse d'utilisation dans les pays à faible et moyen revenu, mais pas dans les pays à haut revenu. Sur les 112 pays et régions affichant une utilisation non nulle de l'application pendant la période de référence et pendant la pandémie, nous avons calculé une réduction médiane de 73,6%.

**Conclusion:**

Les données provenant de l'application fournissent des informations indirectes qui servent à déterminer le nombre d'interventions chirurgicales, et peuvent donc être employées pour suivre en temps réel l'impact de la COVID-19 sur la capacité chirurgicale. Nous avons créé un tableau de bord pour visualiser ces données en continu, ce qui permet aux législateurs d'attribuer des ressources aux secteurs qui en ont le plus besoin.

## Resumen

**Objetivo:**

Determinar si los datos de las aplicaciones móviles para calcular la anestesia asociada a la localización pueden servir como un sustituto cualitativo para evaluar la cantidad de intervenciones quirúrgicas a nivel mundial y, por lo tanto, para medir el impacto de la pandemia de la enfermedad del coronavirus 2019 (COVID-19) en esas intervenciones.

**Métodos:**

Se recopilaron los datos que suministraron los usuarios de la aplicación móvil «Anestesiólogo» durante el periodo comprendido entre el 1.^o^ de octubre de 2018 y el 30 de junio de 2020. Se analizaron a través de RStudio y se generó un gráfico de uso de la aplicación con un promedio variable de 7 días. Se calcularon las reducciones a nivel de país en el uso de la aplicación como un porcentaje de valor basal. Se obtuvieron datos sobre el recuento de los casos de la COVID-19 del Centro Europeo para la Prevención y el Control de las Enfermedades. Se trazaron gráficos sobre el cambio en el uso de las aplicaciones y el recuento de casos de la COVID-19 en varios países y regiones.

**Resultados:**

Un total de 100 099 usuarios de la aplicación en 214 países y territorios suministraron datos. Se observó que el uso de la aplicación se redujo durante los días festivos, los fines de semana y por las noches, en correlación con las fluctuaciones previstas de la cantidad de intervenciones quirúrgicas. Se observó que el inicio de la pandemia generó reducciones sustanciales en el uso de la aplicación. Se registró una fuerte correlación cruzada entre el recuento de los casos de la COVID-19 y las reducciones en el uso de la aplicación en los países de ingresos bajos y medios, pero no en los países de ingresos altos. De los 112 países y territorios que no usaron la aplicación durante el momento basal y durante la pandemia, se calculó una reducción mediana en el uso de la aplicación hasta el 73,6 % del valor basal.

**Conclusión:**

Los datos de la aplicación representan indicadores de la cantidad de intervenciones quirúrgicas y, por lo tanto, se pueden usar para medir en tiempo real el impacto de la COVID-19 en la capacidad quirúrgica. Se ha elaborado un tablero para visualizar estos datos de forma continua, lo que permite a los responsables de formular las políticas asignar recursos a las áreas de mayor necesidad.

## ملخص

الغرض تحديد ما إذا كانت بيانات تطبيق الهاتف المحمول (التطبيق) للآلة الحاسبة للتخدير، والمرتبطة بالموقع، يمكنها أن تكون بمثابة مؤشر نوعي لأحجام الحالات الجراحية العالمية، وبالتالي مراقبة تأثير الإصابة بفيروس كورونا 2019 (كوفيد 19).

الطريقة قمنا بجمع البيانات التي قدمها مستخدمو تطبيق الهاتف المحمول Anesthesiologist (طبيب التخدير) خلال الفترة من 1 أكتوبر/تشرين أول 2018 إلى 30 يونيو/حزيران 2020. كما قمنا بتحليل هذه البيانات باستخدام RStudio، وقمنا بإنشاء مخططات لاستخدام توضح متوسط ​​الحركة لمدة 7 أيام. قم باحتساب حالات الانخفاض في استخدام التطبيق على مستوى البلد كنسبة مئوية من خط الأساس. حصلنا على بيانات عن عدد حالات الإصابة بمرض كوفيد -19 من المركز الأوروبي للوقاية من الأمراض ومكافحتها. قمنا بتخطيط التغيير في استخدام التطبيق وأعداد حالات كوفيد-19 للعديد من البلدان والمناطق.

النتائج إجمالي 100099 من مستخدمي التطبيق في 214 بلدًا وإقليمًا، قاموا بتقديم البيانات. لاحظنا أن استخدام التطبيق قد انخفض خلال الإجازات وعطلات نهاية الأسبوع وأثناء الليل، وهو ما يرتبط بالتقلبات المتوقعة في حجم العمليات الجراحية. لاحظنا أن تفشي الوباء أدى إلى حالات انخفاض ملموسة في استخدام التطبيق. لاحظنا وجود علاقة متبادلة قوية بين عدد حالات الإصابة بكوفيد 19، والانخفاضات في استخدام التطبيق في البلدان منخفضة ومتوسطة الدخل، ولكن ليس في البلدان مرتفعة الدخل. من بين 112 بلدًا وإقليمًا ينعدم فيها استخدام التطبيق أثناء خط الأساس وأثناء الوباء، قمنا باحتساب متوسط ​​الانخفاض في استخدام التطبيق بنسبة 73.6% من خط الأساس.

الاستنتاج توفر بيانات تطبيق الاستنتاج مؤشرًا لأحجام الحالات الجراحية، وبالتالي يمكن استخدامها كمراقب في الوقت الفعلي لتأثير كوفيد 19 على القدرة الجراحية. لقد أنشأنا لوحة تحكم للتصور المستمر لهذه البيانات، مما يسمح لواضعي السياسات بتوجيه الموارد إلى المناطق الأكثر احتياجًا. ****

## 摘要

**目的:**

确定定位麻醉学计算器移动应用程序（以下简称应用）数据是否可以作为全球外科手术量的定性替代指标，从而监测 2019 年全球流行病冠状病毒 (COVID-19) 产生的影响。

**方法:**

我们收集了 2018 年 10 月 1 日至 2020 年 6 月 30 日期间，移动应用程序麻醉师 (Anesthesiologist) 用户的使用数据。我们利用 Rstudio 分析这些数据，并绘制出 7 天内使用应用的移动平均曲线图。我们计算出国家或地区应用使用人数削减数量占基准值的百分比。我们从欧洲疾病预防控制中心了解到新型冠状病毒肺炎病例数。我们绘制了一些国家和地区应用使用人数和新型冠状病毒肺炎病例数量变化图。

**结果:**

214 个国家和地区共有 100,099 名应用用户提供了数据。我们发现，应用使用人数在节假日、周末和晚间有所减少，这与外科手术量的预期波动有关，我们还观察到，全球性流行病爆发导致应用使用人数大幅度下降。我们也注意到，在中低收入国家，新型冠状病毒肺炎病例与应用使用人数减少存在很强的互相关性，而在高收入国家则不存在。基准线和全球性流行病爆发期间，应用使用人数非零的 112 个国家和地区中，我们得出其中位数下降到基准线的 73.6%。

**结论:**

应用数据可以作为外科手术台次的替代指标，因此可用来实时监测新型冠状病毒肺炎对外科手术量的影响。我们设计了一个仪表盘，以便不间断地提供可视化数据，从而决策者可将资源集中于最需要的地区。

## Резюме

**Цель:**

Определить, могут ли данные мобильного приложения калькулятора анестезиологии с привязкой к местоположению служить качественным показателем глобальных объемов хирургических операций и, следовательно, отслеживать влияние пандемии коронавирусного заболевания 2019 г. (COVID-19).

**Методы:**

Авторы собрали данные, предоставленные пользователями мобильного приложения «Анестезиолог» в период с 1 октября 2018 г. по 30 июня 2020 г. Эти данные были проанализированы с помощью программы RStudio, и по ним были построены графики использования приложения для скользящих средних показаний за 7 дней. Сокращение использования приложения на уровне страны было рассчитано в виде процентов от исходного уровня. Данные о количестве случаев COVID-19 авторы получили из Европейского центра по контролю и профилактике заболеваний. Авторы составили график изменения использования приложения в зависимости от количества случаев COVID-19 для нескольких стран и регионов.

**Результаты:**

Данные были получены от 100 099 пользователей приложения из 214 стран и территорий. Было отмечено, что использование приложения сокращалось в праздничные дни, выходные дни и ночью, что соотносится с ожидаемыми колебаниями объема хирургических операций. Также было отмечено, что начало пандемии привело к значительному сокращению использования приложения. Авторы наблюдали сильную взаимную корреляцию между количеством случаев COVID-19 и сокращением использования приложения в странах с низким и средним уровнем дохода, но не в странах с высоким уровнем дохода. По данным для 112 стран и территорий с ненулевым использованием приложения во время исходного уровня и во время пандемии медианное сокращение использования приложения составило до 73,6% от исходного уровня.

**Вывод:**

Данные приложения служат косвенной оценкой количества проводимых хирургических операций и, следовательно, могут использоваться в качестве средства мониторинга воздействия COVID-19 на хирургический потенциал в режиме реального времени. Авторы создали панель индикаторов для непрерывной визуализации этих данных, которая позволяет лицам, формирующим политику, направлять ресурсы в области, где потребности являются наиболее острыми.

## Introduction

The coronavirus disease 2019 (COVID-19) pandemic has caused substantial disruptions to health-care delivery as a result of constrained resources, supply chain interruptions, the need to protect or cover for affected health-care workers, physical distancing and the realities of meeting a surge in demand. Many health-care systems have responded by cancelling or delaying elective surgical procedures.^1^^–^^4^ The downstream impacts of these delays in diagnostic and therapeutic procedural care on public health are unknown. The 2015 Lancet Commission on Global Surgery identified a profound gap in the availability of safe anaesthetic and surgical care in low- and middle-income countries, and estimated that 4.8 billion people lacked access to surgery at baseline before the pandemic.^5^^,^^6^ Although high-income countries are better able to absorb disruptions in surgical care, the effect of the COVID-19 pandemic on unmet surgical needs in low- and middle-income countries could be devastating. The course of recovery to baseline conditions following the pandemic may also be prolonged in low- and middle-income countries as a result of the depletion of health-care resources.

Assessing the volume of global surgical care is notoriously difficult. Prior work in this area has relied on estimations based on modelling and labour-intensive retrospective analysis of data from nations where such information is routinely recorded and available.^5^^,^^7^^–^^9^ Even where such data are available from public health ministries, however, they are not available in real-time and may be limited to health care delivered by government-funded facilities.^10^

**Fig. 1:**
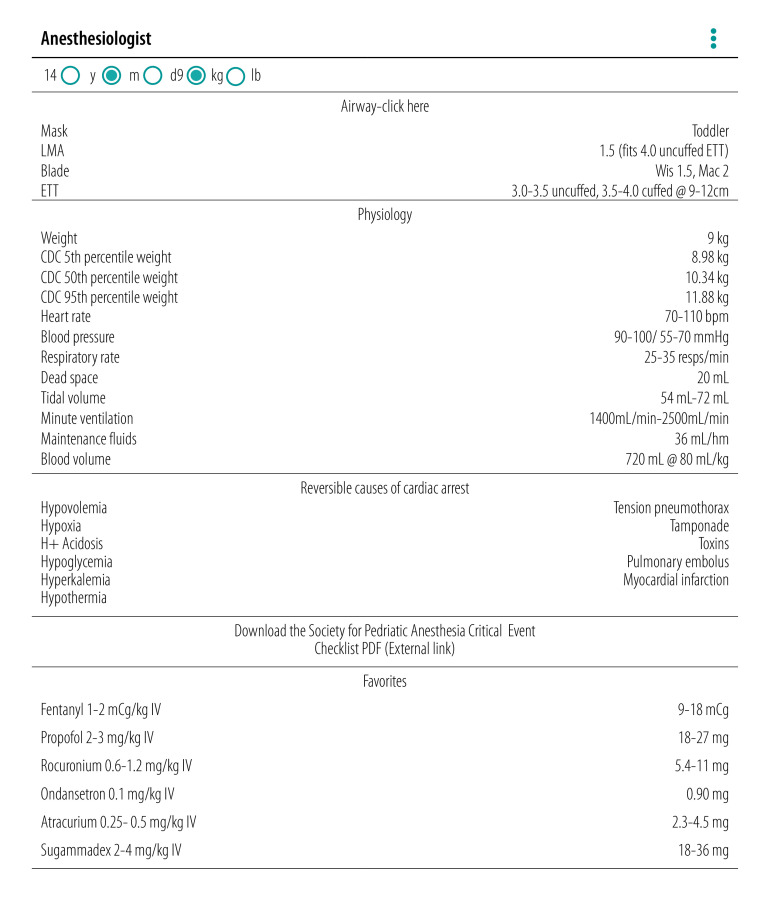
Screen contents of the free Android-platform app Anesthesiologist

We previously developed a free anaesthesia calculator mobile application (app) for the Android platform, called Anesthesiologist.^11^ The app (Fig. 1) has been in use globally since 2011, with over 200 000 users in nearly every country of the world.^12^ The primary users of this app are physician anaesthesiologists and other anaesthesia providers.^12^ The purpose of the app is to provide information fundamental to the practice of anaesthesia, particularly for children, including: physiological parameters by age (e.g. expected weight, blood pressure, heart rate and respiratory rate); airway management information (e.g. endotracheal tube and laryngeal mask airway size); weight-based calculation of appropriate doses for commonly used drugs; and external reference links (e.g. emergency management and peripheral nerve block administration). The app is used by substantially greater numbers of anaesthesia providers in low- and middle-income countries compared with high-income countries.^12^

App: mobile application.

Our aim is to determine whether utilization of the app, aggregated over the large existing international user base, could serve as a real-time qualitative proxy for surgical case volume, and therefore be used to monitor the impact of, and recovery from, the COVID-19 pandemic.

## Methods

### Data sources

Data collection using the app has been described in previous publications.^12^^,^^13^ To summarize, the app provides anaesthesia references and drug calculation capabilities, and following development was made available via the Google Play Store.^11^^,^^14^ After download and the provision of consent, the app records integrated data regarding service utilization, as well as non-compulsory responses to user surveys via Survalytics.^15^ The anonymized information collected includes timestamps from the mobile devices, time zone information, basic demographics, user location (country or dependent territory) from three different sources (global positioning system, internet protocol address^16^ and subscriber identity module country code) and app usage patterns. These data are stored in a cloud database hosted by Amazon Web Services (Seattle, United States of America, USA).^12^^,^^13^ The approach to survey data collection allows users to opt out at any time; unfortunately, this can result in survey fatigue and missing data, impacting the completeness of demographic data collected.^17^ Data analysed here were collected between 1 October 2018 and 30 June 2020 (final full day of data collection). 

We also queried the electronic data warehouses of the University of Washington and the Seattle Children’s Hospital for aggregate surgical case counts for the period 1 October 2018 to 18 April 2020, a time period capturing the relevant drops in surgical case volumes associated with holidays in the USA. We used publicly available data to classify World Health Organization (WHO) regions.^18^ We adopted the publicly available World Bank classification of country income level and region as of July 2020.^19^ Finally, we requested data related to global COVID-19 impact (case and death counts) from the European Centre for Disease Prevention and Control.^20^

### Statistical methods

We analysed raw data in R using RStudio v3.6.2 (R Foundation, Vienna, Austria);^21^^,^^22^ full R code and raw data are available on request. We excluded data containing timestamps before or following the period of interest, including a small number of observations (280) with invalid timestamps. We calculated time-series data from incoming individual data points (including logged app uses and in-app navigation) from all users. Individual users may have used the app in more than one country or dependent territory, as defined by the International Organization for Standardization ISO-3166 α-2 country code associated with the data point; in this case, we assigned the user-level country code in which the majority of uses occurred.

We performed change point detection in time-series data using the cpm package.^23^ For clarity of presentation, we generated the app use plots using a 7-day moving average to mitigate the routine effect of app use reductions during weekends (available in data repository).^24^ For all countries or territories with non-zero user counts during both the baseline period (1 September 2019–1 November 2019) and the period spanning the most recently available data (25–30 June 2020), we calculated (i) the country-level reduction in app use, which is the mean daily count of recent app use as a percentage of mean daily count of baseline use, and (ii) the median value of these percentage reductions. We generated a map of the estimated global impact of the pandemic on surgery volume using the tmap package for R.^25^

### Ethics approval and manuscript preparation

The study was reviewed and approved by the Emory University Institutional Review Board (study no. 00082571), and there is a reliance agreement in place with the University of Washington Institutional Review Board. The approval includes a waiver of written informed consent. Participants gave electronic consent anonymously before participating in any data collection. The app is a medical device that falls into the category of enforcement discretion as per the United States Food and Drug Administration.^26^

## Results

### Demographics

From 1 October 2018 to 30 June 2020, we collected and analysed 4 827 263 data points from 100 099 unique users in 214 countries and territories (Box 1). Approximately half of the users (50.9%; 50 989/100 099) completed or partly completed the survey on their position and the characteristics of their practice; we summarize the provider demographics of these users in Table 1. The majority of users who responded to the survey were anaesthesia providers: physicians, certified registered nurse anaesthetists or anaesthesiologist assistants. As identified in a previous publication, anaesthesia officers practising in low-income countries may have identified as being technically trained in anaesthesia or were otherwise self-identified.^12^ We noted a large variation with respect to self-reported elements of the practice environment; however, the distribution of participant characteristics was consistent with previous findings.^12^ We provide counts of the number of unique users per country or territory that provided data during the study period in Box 1.

Box 1 **Number of unique users of Anesthesiologist app per country or territory, 1 October 2018–30 June 2020**

India: 12 374; United States of America: 4259; Germany: 4092; Russian Federation: 3630; Indonesia: 3352; Italy: 3304; Mexico: 2888; Pakistan: 2844; Brazil: 2155; Poland: 2052; Turkey: 2003; Egypt: 1709; Algeria: 1679; Spain: 1625; Colombia: 1520; Malaysia: 1353; France: 1267; Ethiopia: 1249; Ukraine: 1218; Nigeria: 1204; Kenya: 1156; Romania: 1136; Philippines: 1112; Iran (Islamic Republic of): 1097; Sudan: 1085; United Republic of Tanzania: 1067; Peru: 1032; Ghana: 1031; Yemen: 1019; Portugal: 1010; Libya: 997; Iraq: 961; United Kingdom: 926; Saudi Arabia: 924; South Africa: 903; Argentina: 732; Cuba: 724; Ecuador: 720; Bangladesh: 705; Democratic Republic of the Congo: 678; Belarus: 628; Viet Nam: 614; Netherlands: 610; Afghanistan: 567; Morocco: 565; Hungary: 561; China: 535; Chile: 507; Czechia: 507; Bolivia (Plurinational State of): 505; Israel: 468; Nepal: 462; Slovenia: 453; Madagascar: 450; Australia: 446; Croatia: 437; Austria: 436; Cameroon: 434; Venezuela (Bolivarian Republic of): 434; Belgium: 420; Uzbekistan: 392; Canada: 389; Bulgaria: 386; Greece: 363; Tunisia: 360; Rwanda: 354; Serbia: 350; Myanmar: 327; Uganda: 315; Kazakhstan: 310; Switzerland: 305; Syrian Arab Republic: 298; Côte d’Ivoire: 297; Dominican Republic: 295; Slovakia: 272; Sweden: 272; Thailand: 270; Jordan: 264; Somalia: 256; Republic of Korea: 245; Mali: 241; Zimbabwe: 236; United Arab Emirates: 230; Georgia: 221; Angola: 220; Bosnia and Herzegovina: 199; Burundi: 198; Lithuania: 197; Zambia: 197; Paraguay: 194; Ireland: 187; Norway: 186; Sri Lanka: 178; Azerbaijan: 175; Lao People's Democratic Republic: 173; Haiti: 161; Lebanon: 161; Kuwait: 154; Cambodia: 146; Liberia: 145; El Salvador: 144; Niger: 142; Latvia: 139; West Bank and Gaza Strip: 138; Taiwan, China: 135; North Macedonia: 130; Mauritius: 129; Papua New Guinea: 124; Chad: 120; Nicaragua: 117; Republic of Moldova: 117; Mozambique: 114; Oman: 112; Senegal: 110; Honduras: 104; Tajikistan: 98; Fiji: 97; Albania: 91; Trinidad and Tobago: 91; Burkina Faso: 90; Guatemala: 90; Turkmenistan: 89; Mongolia: 87; Japan: 85; Qatar: 84; Namibia: 81; Guinea: 78; Guyana: 76; Singapore: 76; China, Hong Kong SAR: 74; Kyrgyzstan: 74; Uruguay: 74; Panama: 73; Denmark: 72; Estonia: 71; Jamaica: 71; Finland: 69; Sierra Leone: 68; Armenia: 67; New Zealand: 67; Congo: 64; Gambia: 62; Malawi: 62; Kosovo: 56; Benin: 55; Costa Rica: 54; Mauritania: 54; Cyprus: 48; Puerto Rico: 45; South Sudan: 45; Bahrain: 39; Botswana: 38; Djibouti: 37; Lesotho: 36; Comoros: 33; Bhutan: 32; Gabon: 31; Togo: 29; Montenegro: 28; Maldives: 26; Luxembourg: 21; Belize: 20; French Réunion: 19; Seychelles: 17; Suriname: 17; Bahamas: 16; Malta: 16; Sao Tome and Principe: 15; Barbados: 14; Iceland: 14; Antigua and Barbuda: 13; Central African Republic: 13; Solomon Islands: 13; Eswatini: 13; Timor-Leste: 12; Equatorial Guinea: 11; Cabo Verde: 10; Tonga: 9; Brunei Darussalam: 8; New Caledonia: 8; Guadeloupe: 7; Guinea-Bissau: 7; China, Macao SAR: 6; Tuvalu: 5; Vanuatu: 5; Aruba: 4; Martinique: 4; Monaco: 4; Anguilla: 3; Cayman Islands: 3; Kiribati: 3; Micronesia (Federated States of): 3; Saint Lucia: 3; Saint Vincent and the Grenadines: 3; American Samoa: 2; Eritrea: 2; French Polynesia: 2; Mayotte: 2; Åland Islands: 1; Andorra: 1; Bermuda: 1; Cook Islands: 1; Curaçao: 1; Dominica: 1; Faroe Islands: 1; Greenland: 1; Grenada: 1; Isle of Man: 1; Liechtenstein: 1; Palau: 1; Saint Martin (French part): 1; Samoa: 1; San Marino: 1; United States Virgin Islands: 1.

App: mobile application; SAR: Special Administrative Region.Note: A small number of users (44) had country codes that were not standard ISO 3166 α-2 codes, meaning that their country of origin could not be reliably determined.

**Table 1:** Users of Anesthesiologist app and properties of their practice,^a^ 1 October 2018 to 30 June 2020

Properties of app user and their practice	No. (%)
**User characteristics, if provided (*n* = 50 989)^b^**	
Physician attending or consultant	13 198 (25.9)
Physician resident, fellow or registrar	10 904 (21.4)
Certified registered nurse anaesthetist or anaesthesiologist assistant	12 748 (25.0)
Certified registered nurse anaesthetist or trainee anaesthesiologist assistant	2 367 (4.6)
Technically trained in anaesthesia	1 408 (2.8)
Anaesthesia technician	3 089 (6.1)
Medical student	2 282 (4.5)
Nurse	1749 (3.4)
Paramedic emergency medical technician	1 089 (2.1)
Respiratory therapist	381 (0.7)
Pharmacist	447 (0.9)
Other medical practitioner	817 (1.6)
Not medical practitioner	510 (1.0)
**Practice model, if provided (*n* = 22 315)^b^**	
Physician only	6 835 (30.6)
Physician supervised, anaesthesiologist onsite	9 300 (41.7)
Physician supervised, non-anaesthesiologist physician onsite	2 077 (9.3)
Physician supervised, no physician onsite	1 319 (5.9)
No physician supervision	1 519 (6.8)
Not an anaesthesia provider	1 265 (5.7)
**Practice type, if provided (*n* = 23 586)^b^**	
Private clinic or office	4 638 (19.7)
Local health clinic	2 166 (9.2)
Ambulatory surgery centre	1 525 (6.5)
Small community hospital	2 933 (12.4)
Large community hospital	6 413 (27.2)
Academic department or university hospital	5 911 (25.1)
**Practice size, if provided (*n* = 28 090)^b^**	
Single practitioner for a large area	7 890 (28.1)
One of several practitioners in the area	5 445 (19.4)
Group practice size (members)	
1–5	3 710 (13.2)
> 5–10	2 930 (10.4)
> 10–25	2 731 (9.7)
> 25–50	2 219 (7.9)
> 50	3 165 (11.3)

App: mobile application.

^a^ Mean length of practice, 13.2 years (standard deviation: 13.4 years).

^b^ Completion of any part of the survey is not compulsory for users of the app.

### Impact of inherent factors 

**Fig. 2:**
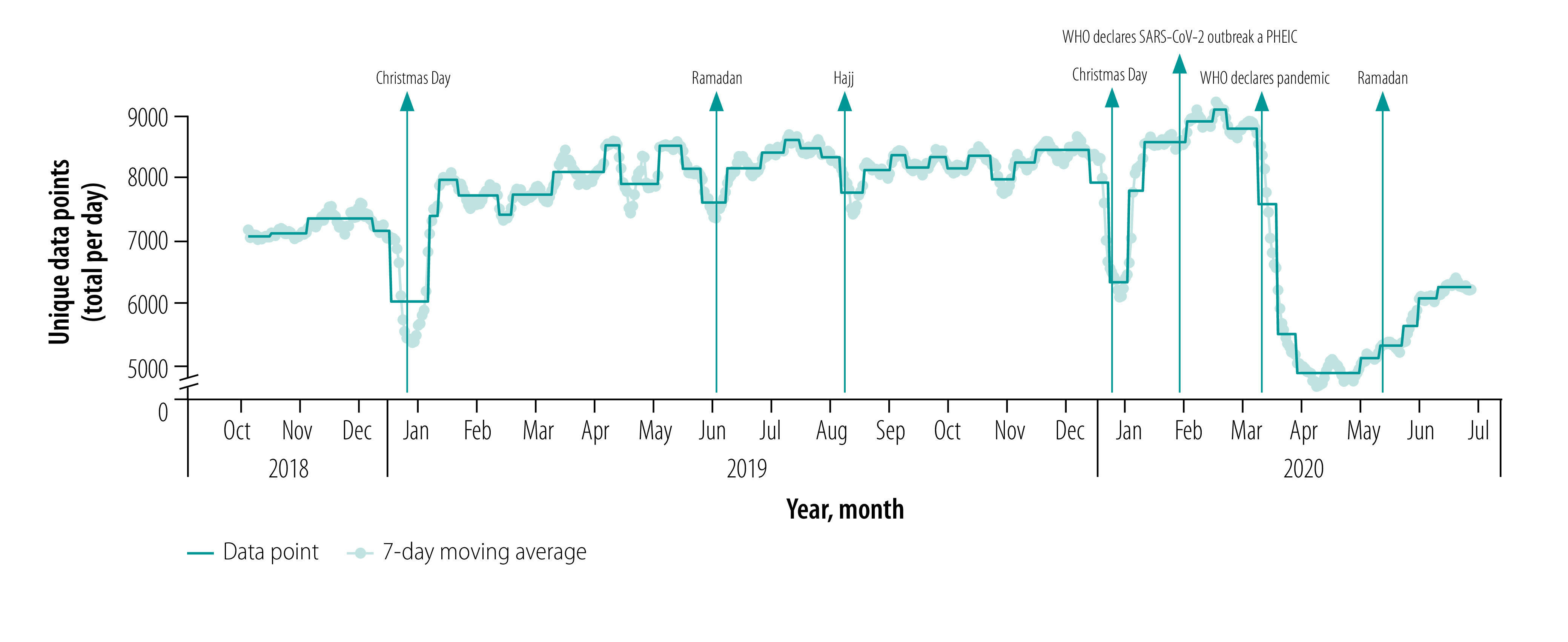
Time-series data depicting Anesthesiologist app use for all users, 1 October 2018–30 June 2020

**Fig. 3:**
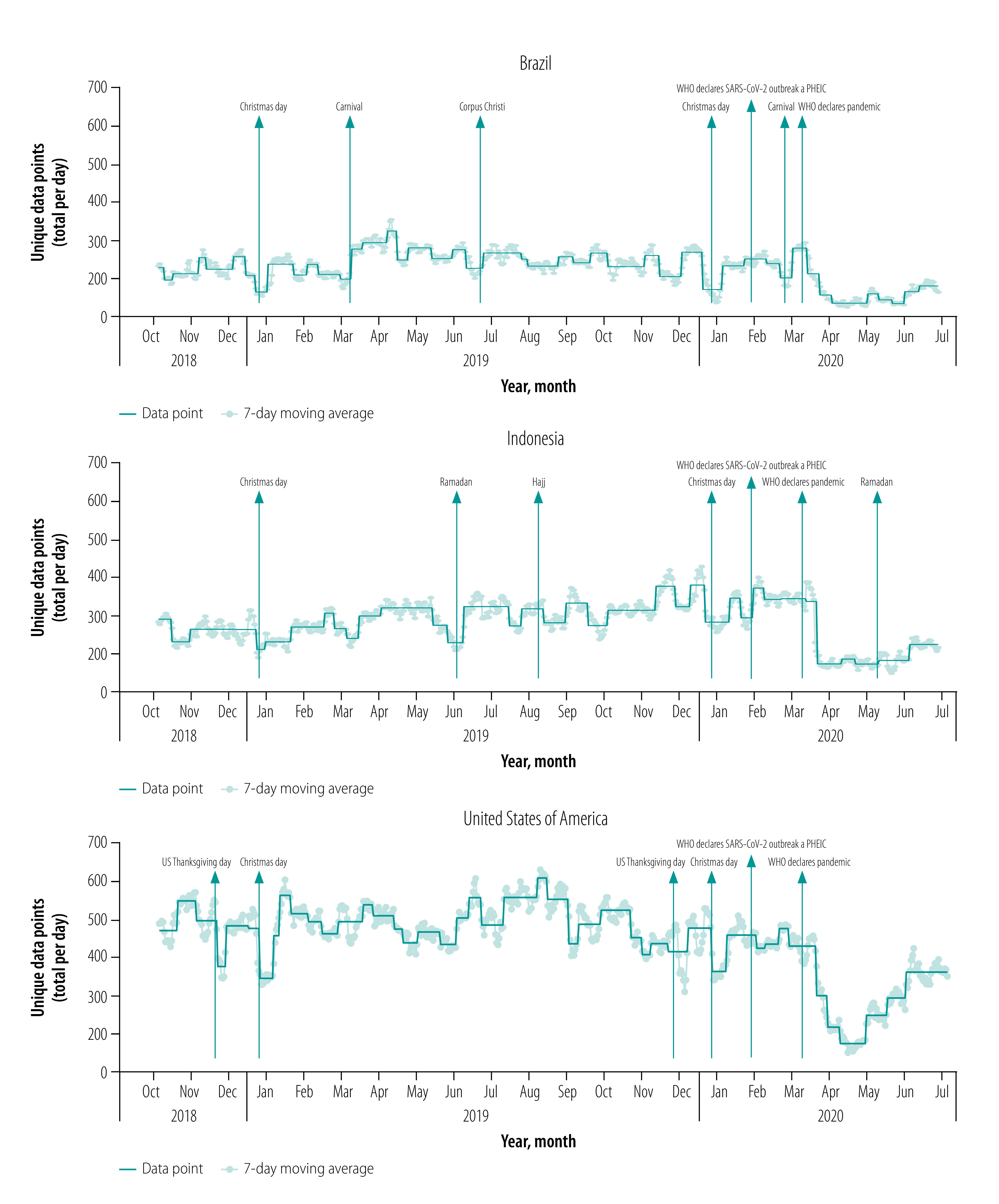
Time-series data depicting Anesthesiologist app use for users in Brazil, Indonesia and the United States of America, 1 October 2018–30 June 2020

We demonstrate in Fig. 2 that there was consistent provision of data by users over the study period. Our country- or region-specific data also indicate large reductions in app use coinciding with major holidays,^27^^–^^30^ and hence anticipated reductions in surgical case volumes. In the USA (Fig. 3), we observed reductions in app use during the period around Thanksgiving Day (i.e. the fourth Thursday of November) and Christmas Day. This finding is consistent with case volume data obtained from the University of Washington Medical Center and Seattle Children’s Hospital (data repository),^24^ and has been previously described in the literature. In Indonesia, in which the majority of the population self-identify as Muslim (Fig. 3), we observed large reductions in app use during the month of Ramadan and a smaller decrease around Christmas Day, but not around Thanksgiving Day. We also noted reductions in app use around the time of Hajj as well as Ramadan in aggregated data from 38 Muslim-majority countries (data repository).^24^ In Brazil (Fig. 3), which has a large app user base, we detected a notable decrease in app use during the Carnival celebration in 2019 and 2020 and the Corpus Christi celebration in 2019. 

App: mobile application, PHEIC: public health emergency of international concern, WHO: World Health Organization.

Note: We performed change point detection in time-series data using the cpm package.^23^

App: mobile application, PHEIC: public health emergency of international concern, US: United States of America, WHO: World Health Organization.

Note: We performed change point detection in time-series data using the cpm package.^23^

We also observed an expected variation in app use by the day of the week, consistent with known data demonstrating that surgical case volumes are highest during the middle of the week and much lower over weekends (data repository).^24^^,^^28^ The expected diurnal variation in app use, peaking between 07:00 and 09:00 local time and with minimal use between midnight and 06:00 local time (data repository),^24^ was also evident.

### Impact of COVID-19

**Fig. 4:**
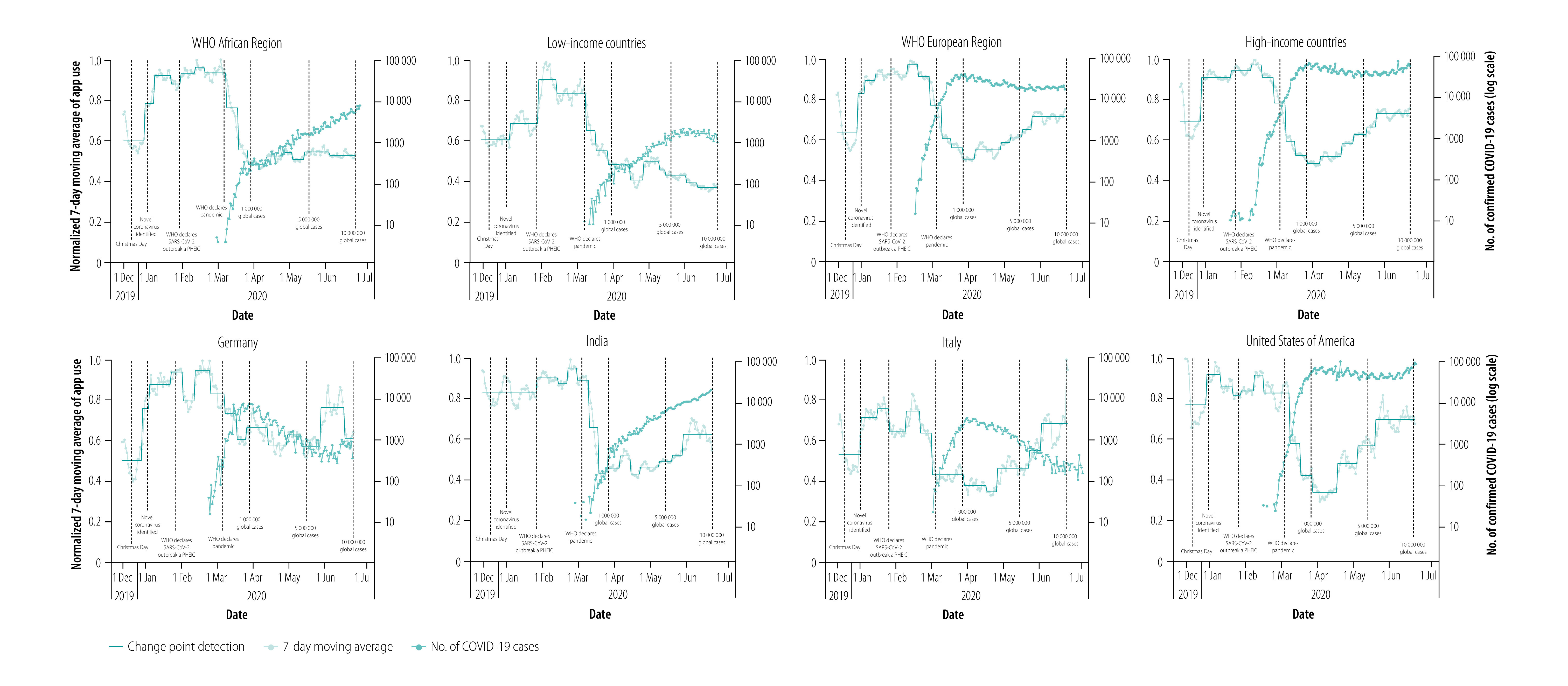
Association between use of the Anesthesiologist app, a proxy for surgical case volumes, and COVID-19 cases, December 2019–June 2020

We illustrate the impacts of COVID-19 on app use in Fig. 4. Notably, all regions demonstrated steep declines in app use following the WHO declaration of global pandemic status. Some recovery from nadir is apparent, although this recovery in app use varies widely between countries and/or regions.

App: mobile application; COVID-19: coronavirus disease 2019; PHEIC: public health emergency of international concern, WHO: World Health Organization.

Note: We performed change point detection in time-series data using the cpm package.^23^ Data points were normalized after calculation of the seven-day moving average by dividing each data point by the maximum data point in the set of values.

We further used this data to illustrate variability in app use relative to counts of COVID-19 cases. Overall, app use declined as COVID-19 cases increased during the study period (Fig. 4). We found that in countries of the WHO African Region, the profound decrease in app use plateaued by around the beginning of April 2020, and did not demonstrate any recovery by the end of the study period. In low-income countries, app use briefly plateaued before continuing on a downwards trend. In countries of the WHO European Region, the major reduction in app use in March 2020 was concordant with the WHO pandemic declaration. From the nadir in April 2020, app use rebounded but not to baseline levels. Similar findings were seen in high-income countries: reductions in app use rebounded from a minimum in April 2020. In Germany and Italy, decreases in app use were measured in February 2020, weeks before the WHO pandemic declaration. We observed further reductions in app use after the pandemic declaration, with the subsequent recovery to near baseline in Italy but depressed from baseline in Germany. However, we note that the degree of depression in app use in Germany was not as large as the degree of depression in Italy. In India and the USA decreases in app use followed the WHO declaration and were abrupt. App use in both countries has trended upwards from a minimum, although this recovery was slower in India compared with in the USA.

We generated a map of the global impact of COVID-19 on app use (available in the data repository),^24^ which indicated a widespread reduction. Of the 214 countries and territories included in the study (Box 1), users in 112 reported use during both the periods (baseline and recent). We calculated the median reduction in app use of these 112 countries and territories as 73.6% of baseline (inter-quartile range, 57.1–96.0%), with the highest reduction in app use to 18.6% (5.21/28.07) of baseline in Burundi (Table 2). Of the 102 countries and territories with no app use during the baseline or recent periods, and therefore not included in the median reduction calculation, 38 were classified as high income, 21 as upper-middle income, 23 as lower-middle income and 13 as low income; 7 did not have a World Bank income-level classification. We observed a mixed relationship between overall reduction in app use and COVID-19 case count at the end of the study period (data repository).^24^ In middle-income countries, higher case counts did correlate with lower app use; however, no relationship was observed in high-income countries. We measured the greatest reduction in app use in low-income countries (data repository).^24^ When examining the cross-correlation function of COVID-19 case count on a day-to-day basis versus daily app use counts in individual regions and countries, we observed a strong inverse correlation between these two time series at very low lags (data repository).^24^

**Table 2:** Reduction in average daily Anesthesiologist app use per country or dependent territory^a^ as a result of the coronavirus pandemic, 25–30 June 2020

Country or territory	Average daily app use (counts)		Change in app use as percentage of baseline^c^
	Baseline^b^	25–30 June 2020	
Afghanistan	23.8	9.6	40.3
Algeria	85.9	78.0	90.8
Angola	23.1	11.1	47.9
Argentina	55.2	30.9	55.9
Armenia	12.6	5.9	46.6
Australia	39.9	28.5	71.5
Austria	27.9	14.3	51.1
Azerbaijan	18.8	7.9	41.7
Bahrain	9.7	10.0	103.2
Bangladesh	50.8	24.6	48.3
Belarus	54.2	22.8	42.0
Belgium	40.5	40.0	98.7
Bolivia (Plurinational State of)	57.7	35.2	61.0
Bosnia and Herzegovina	20.2	12.1	60.3
Brazil	245.9	167.1	67.9
Bulgaria	46.7	30.4	65.2
Burkina Faso	13.4	7.6	56.6
Burundi	28.1	5.2	18.6
Cameroon	30.6	23.8	77.8
Canada	26.7	10.4	38.7
Chile	42.9	35.9	83.6
China	37.6	37.6	99.9
Colombia	127.8	118.6	92.8
Côte d’Ivoire	32.7	27.7	84.7
Croatia	63.4	40.4	63.7
Cuba	41.5	25.0	60.2
Cyprus	10.2	10.5	102.7
Czechia	41.6	24.7	59.5
Democratic Republic of the Congo	40.6	28.1	69.4
Dominican Republic	29.5	28.6	97.1
Ecuador	100.5	68.4	68.1
Egypt	118.5	88.1	74.4
Ethiopia	75.0	29.1	38.9
Fiji	17.0	26.6	156.1
France	91.2	51.3	56.2
Georgia	19.0	17.6	92.6
Germany	210.3	162.1	77.1
Ghana	86.5	111.9	129.3
Greece	42.4	41.6	98.1
Guinea	8.1	8.7	107.4
Guyana	19.7	7.9	39.9
Haiti	18.3	23.7	129.5
Hungary	51.0	36.9	72.3
India	899.5	578.3	64.3
Indonesia	312.8	213.4	68.2
Iran (Islamic Republic of)	51.4	34.0	66.1
Iraq	31.4	19.3	61.4
Ireland	22.1	73.5	332.6
Israel	56.2	61.2	109.0
Italy	230.1	372.1	161.7
Jamaica	16.7	5.6	33.3
Jordan	19.1	36.1	189.2
Kazakhstan	16.7	17.0	101.9
Kenya	206.5	118.7	57.5
Kosovo	9.8	5.6	57.1
Kuwait	11.8	8.9	74.8
Lao People's Democratic Republic	19.4	16.6	85.7
Latvia	21.2	18.1	85.2
Lebanon	14.8	10.6	71.7
Libya	88.8	75.9	85.5
Lithuania	19.2	16.0	83.5
Madagascar	18.6	9.6	51.8
Malawi	20.6	6.1	29.8
Malaysia	166.4	186.6	112.1
Mali	13.1	11.7	89.4
Mauritius	14.1	14.4	101.6
Mexico	282.3	223.8	79.3
Mongolia	13.3	19.7	147.9
Morocco	29.3	22.1	75.4
Myanmar	15.1	11.6	76.5
Namibia	9.4	29.2	310.3
Nepal	27.6	34.3	124.0
Netherlands	44.2	38.9	87.9
Nicaragua	7.7	7.0	90.3
Nigeria	101.0	54.9	54.4
Norway	17.1	9.9	57.7
Pakistan	194.6	87.9	45.2
Papua New Guinea	14.7	7.5	51.0
Paraguay	17.0	16.5	97.1
Peru	128.7	53.5	41.6
Philippines	166.2	128.6	77.4
Poland	103.4	70.1	67.8
Portugal	115.1	84.4	73.3
Republic of Korea	14.7	8.4	57.0
Romania	88.0	83.6	95.0
Russian Federation	319.4	173.0	54.2
Rwanda	32.6	31.2	95.7
Saudi Arabia	94.7	55.0	58.1
Serbia	21.9	15.9	72.7
Slovakia	26.5	24.9	93.8
Slovenia	43.8	37.1	84.9
South Africa	114.4	67.5	59.0
Spain	138.5	122.5	88.5
Sri Lanka	22.7	8.9	39.1
Sudan	91.0	30.9	34.0
Sweden	19.0	12.1	63.9
Switzerland	16.0	20.4	128.1
Syrian Arab Republic	14.7	12.7	86.3
United Republic of Tanzania	82.0	129.8	158.3
Trinidad and Tobago	15.7	13.4	85.4
Turkey	101.1	126.2	124.9
Uganda	67.8	30.6	45.1
Ukraine	59.3	41.3	69.6
United Arab Emirates	23.4	16.1	68.8
United Kingdom	82.7	35.6	43.1
United States of America	489.1	361.9	74.0
Venezuela (Bolivarian Republic of)	41.1	39.8	96.8
Viet Nam	32.3	41.1	127.2
West Bank and Gaza Strip	9.1	10.3	113.4
Yemen	56.2	39.3	70.0
Zambia	16.0	32.3	201.6
Zimbabwe	22.1	9.4	42.3

App: mobile application.

^a^ We only included the 112 countries or territories with non-zero app use during both periods in the median reduction calculation.

^b^ 1 September to 1 November 2019.

^c^ Note that average daily user counts were calculated to several decimal places, hence the apparent rounding errors in percentage reduction calculations.

## Discussion

Many people in low- and middle-income countries are already without adequate access to safe anaesthetic and surgical care at baseline.^6^ Here, we have shown that the impact of the COVID-19 pandemic on the use of our app, a proxy for surgical case volumes, has exacerbated this burden, especially in low-income countries. Although recovery of app use has been substantial in high-income countries, that recovery has yet to be realized in low-income environments. 

The benefits and challenges associated with the collection of health-care data using mobile technology have been discussed in previous publications.^31^^,^^32^ The benefits include a decentralized approach to the collection of data from the 95% of the global population living in an area covered by, and subscribed to, a mobile cellular service.^33^ A total of 5.5 billion mobile phone subscriptions were recently reported in low- and middle-income countries, representing nearly 92 subscriptions per 100 inhabitants.^34^ Challenges include the dissemination of specific applications, the types of data that can be collected, the trade-off between apps that have clinical utility and the data that can be gleaned from the use of these apps, the use of multiple platforms (e.g. Android, iOS), and the analysis and interpretation of stochastic app use data.

A specific strength of our work is the practical use of the app from which data were gathered and analysed. Users download and use the app for the clinical care of patients. The app has never been advertised or its use encouraged via notifications or other mechanisms, meaning that use of the app reflects stochastic clinical care events. 

This same stochasticity highlights a limitation of the work, however; data from individual regions or countries with a small user base reduce the confidence we can assign to the association between app use time-series data and surgical case volume. By excluding many more high- and middle-income countries than low-income countries with zero app use during the relevant periods in our calculation of median app use reduction, we have probably underestimated the global impact of COVID-19 on surgical case volumes.

Another important limitation is that the app is used primarily for the care of paediatric cases (about 75% of app uses are for patients aged 12 years and younger), and this predominance may drive greater use of the app in low- and middle-income countries where (i) subspecialty training in paediatric anaesthesia is less prevalent compared with in high-income countries; and (ii) as much as 50% of the population in low- and middle-income countries may be younger than 16 years. Notably, these needs in low- and middle-income countries are not trivial: 1.7 billion children lack adequate access to surgical care and an estimated 85% of children will need surgical care by the age of 16 years.^35^ Further, given the differential impact of COVID-19 in younger versus older populations, and the proportion of elective versus non-elective surgery in paediatric patients, a greater degree of paediatric surgery may be seen compared with surgery for adults. Conversely, app utilization patterns may be relatively less impacted in high-income countries that have dedicated paediatric hospitals. 

Finally, we acknowledge that many factors may drive changes in patterns of app use, and hence the relationship between app use and surgical case volume for a given country or region. For example, users are more likely to consult the app during emergencies,^36^ meaning that app use during weekends (when a greater proportion of surgical cases are emergencies) is proportionally greater than would be expected based on actual surgical case volumes. Widespread changes in the distribution and active use of the app (e.g. increased adoption or the loss of users to alternative apps) would require our analysis to be adjusted for changes in the size of the user base. Individual users could also skew the data by downloading and activating the app with no intention of using it; this might cause local distortions but would require a concerted effort to impact the broader trends seen in the data. Higher-income countries may also have benefitted from the resources (e.g. testing kits and personal protective equipment) to continue with elective procedures safely, despite rising COVID-19 case numbers. 

In conclusion, we present a real-time qualitative monitor of the impact of COVID-19 on global surgical volumes, particularly in low- and middle-income countries. Combined with other information sources, our app provides governments, global health organizations and philanthropic groups access to data providing markers of recovery – or otherwise – of surgical capacity, as well as the opportunity to direct resources to the areas of greatest need. To ensure the ongoing accessibility of this information, we have developed a near real-time dashboard (http://globalcases.info). Longer term, our app could be combined with other data to assist with measurement of global surgical capacity as part of the Global Surgery 2030 initiative. 
